# Fine-grained lung cancer object detection via dilated reparameterization and explicit positional gating optimization

**DOI:** 10.1038/s41598-026-58090-0

**Published:** 2026-06-20

**Authors:** Yitao Wu, Ziqiong He, Su Zhang, Chen Liang, Chen Zhang, Xue Yang, Yizhe Zhang, Shuwen Wang, Kaiyu Cai

**Affiliations:** 1 Department of Respiratory and Critical Care Medicine, Jinjiang Municipal Hospital, Quanzhou, Fujian People’s Republic of China; 2 Research Institute of General Surgery, Jinling Hospital, Affiliated Hospital of Medical School, Nanjing University, Nanjing, China; 3 Department of Rheumatology, Nanping First Affiliated Hospital of Fujian Medical University, Nanping, People’s Republic of China; 4 The Affiliated Jiangning Hospital of Nanjing Medical University, Nanjing, People’s Republic of China; 5 Department of General Surgery, The Second Affiliated Hospital of Fujian Medical University, QuanZhou, 362000 Fujian Province People’s Republic of China; 6 Department of Cardiology, Nanping First Affiliated Hospital of Fujian Medical University, Nanping, People’s Republic of China

**Keywords:** Lung cancer detection, YOLO, Fine-grained classification, Reparameterization, Attention mechanism, Dynamic upsampling, Cancer, Computational biology and bioinformatics, Mathematics and computing

## Abstract

Early detection and precise fine-grained radiologic classification of lung cancer are crucial for formulating clinical treatment plans. However, existing computer-aided diagnosis (CAD) systems are mostly limited to binary nodule classification or treat radiologic feature classification and clinical TNM staging as isolated tasks. To address technical challenges such as drastic scale variations of tumors, subtle inter-class differences, and complex anatomical backgrounds, this paper proposes an end-to-end fine-grained lung cancer object detection algorithm based on an improved YOLO architecture. The proposed model accomplishes a complex 7-class detection task within a unified framework, encompassing normal tissue, three major non-small cell lung cancer (NSCLC) subtypes, and specific anatomical locations combined with TNM stages. Specifically, a Dilated Reparameterization Block (C3k2_DRB) is designed in the backbone to effectively enlarge the receptive field via multi-scale dilated convolutions, enhancing feature extraction for multi-scale tumors without introducing inference overhead. Meanwhile, an Explicit Positional Gating Optimization attention mechanism (C2PSA_EPGO) is introduced to adaptively focus on the most discriminative lesion textures and suppress irrelevant background noise. Furthermore, a dynamic upsampling strategy (DySample) is employed in the feature fusion stage to achieve content-aware spatial alignment, maximally preserving the morphological boundary details of the lesions. Experimental results on a complex lung cancer dataset comprising 1886 images demonstrate that the improved model achieves 97.6% Precision and 99.2% mean Average Precision (mAP@0.5) while maintaining extremely low parameters (2.46 M) and computational cost (6.3 GFLOPs). Compared with mainstream object detection algorithms, the proposed method exhibits significant advantages in localization precision and fine-grained classification performance, demonstrating the potential of the proposed architectural enhancements for complex, fine-grained medical vision tasks.

## Introduction

Lung cancer is one of the most prevalent and lethal malignancies worldwide, with non-small cell lung cancer (NSCLC) accounting for the vast majority of cases^1^. Early detection and precise diagnosis are crucial for improving the five-year survival rate of patients. In clinical practice, formulating an accurate treatment plan—such as targeted therapy, immunotherapy, or surgical resection—relies not only on the precise localization of lung lesions but also on the clear identification of radiologic subtypes (e.g., imaging features indicative of adenocarcinoma, squamous cell carcinoma, large cell carcinoma) and TNM clinical staging (tumor size, lymph node involvement, and metastasis). Therefore, developing an intelligent computer-aided diagnosis (CAD) system capable of simultaneously achieving lesion localization, radiologic feature classification, and clinical staging holds significant clinical value^2^.

Recently, deep learning-based object detection algorithms have made remarkable progress in medical image analysis^3^. For instance, Wang et al.^4^ demonstrated that improved YOLO architectures can significantly enhance the screening efficiency of pulmonary nodules. Furthermore, Coudray et al.^5^ proved the immense potential of deep learning in classifying NSCLC subtypes and predicting mutations. Despite these advancements, existing CAD systems are mostly limited to the binary classification of benign and malignant nodules or perform subtyping as isolated classification tasks. Applying object detection to complex, end-to-end lung cancer tasks that simultaneously include fine-grained subtyping and TNM staging presents substantial technical challenges.

First, lung tumors of different stages and subtypes vary drastically in scale and morphology. Although traditional multi-scale strategies like Feature Pyramid Networks (FPN)^6^ and hierarchical Vision Transformers^7^ have been widely used, and Ding et al.^8^ proposed large-kernel and dilated convolution strategies to effectively expand receptive fields for broad perception, it remains difficult for standard feature extraction networks, even classic medical architectures like U-Net^9^, to balance global context and local textures effectively without introducing massive computational overhead. Second, medical images are often accompanied by complex anatomical backgrounds, and the inter-class variance between different radiologic feature classification (such as adenocarcinoma and squamous cell carcinoma) is extremely small, despite the distinct prognostic values hidden in their texture features^10^. While classical static attention mechanisms^11^ and recent advancements like the Pointwise Spatial Attention (PSA) introduced by Wang et al.^12^ have shown promise in reducing computational redundancy while focusing on salient features, they often struggle to adapt to the highly variable appearances of advanced-stage tumors, making the model susceptible to background noise. Finally, during the feature fusion stage, traditional upsampling methods (such as nearest-neighbor interpolation) tend to lose the edge and contour details of the lesions. As Liu et al.^13^ pointed out in their work on dynamic upsampling, fixed upsampling operators lack content-awareness, often leading to degraded localization accuracy, particularly for tumors with irregular boundaries.

To address these issues, this paper proposes a fine-grained lung cancer object detection algorithm based on an improved YOLO architecture. The proposed algorithm can accomplish a complex 7-class detection task within an end-to-end framework, encompassing normal tissue, cancer cells, three major NSCLC subtypes, and specific anatomical locations combined with TNM stages.

Specifically, we introduce the Dilated Reparameterization Block (DRB) into the backbone network to construct a novel C3k2_DRB structure. This module effectively enlarges the receptive field through multi-scale dilated convolutions, enhancing the model’s ability to capture features of tumors of varying sizes while maintaining high inference efficiency via structural reparameterization. Furthermore, to tackle complex backgrounds and subtle radiologic differences, we introduce an Explicit Positional Gating Optimization (EPGO) attention mechanism in the deep layers of the backbone, constructing the C2PSA_EPGO module. This allows the network to adaptively focus on the most discriminative lesion areas and suppress irrelevant tissue interference. In the neck feature fusion network, we introduce a dynamic upsampling module (DySample) to replace traditional linear interpolation. By learning the spatial offsets of point sampling, DySample achieves dynamic alignment of feature maps, maximally preserving the boundary details of the tumor and significantly improving localization precision.

The main contributions of this paper are summarized as follows:

- We construct a clinically oriented, fine-grained detection task that breaks through the limitations of traditional binary nodule classification. It achieves a highly challenging 7-class detection covering radiologic subtypes, anatomical locations, and TNM clinical stages, exploring a unified algorithmic framework for fine-grained medical object detection.
- We introduce the C3k2_DRB and C2PSA_EPGO feature extraction modules. By combining multi-scale dilated reparameterization with a dynamic gated attention mechanism, we effectively address the issues of large scale variations and small inter-class differences in lung cancer lesions, enhancing the feature representation capability of the model.
- We introduce the DySample dynamic feature fusion strategy. Without significantly increasing the computational burden, it optimizes the multi-scale feature upsampling process, strengthening the model’s perception of tumor edge details.
- The improved model demonstrates superior experimental performance. On the constructed complex lung cancer imaging dataset, our method significantly outperforms mainstream object detection algorithms in terms of mean Average Precision (mAP), indicating that the targeted architectural modifications successfully address multi-scale and fine-grained challenges in a proof-of-concept setting.

## Related work

### Computer-aided diagnosis for lung cancer

The integration of deep learning into computer-aided diagnosis (CAD) systems has revolutionized the workflow of radiologic analysis^2,3^. Early approaches in lung cancer detection heavily relied on hand-crafted features and traditional machine learning algorithms, which were later significantly propelled by public benchmarks such as the LIDC-IDRI database^14^ and the LUNA16 challenge^15^. For instance, Wang et al.^4^ utilized shape constraint models for pulmonary nodule detection in CT images. With the advent of deep learning, researchers began to explore more complex clinical tasks. Coudray et al.^5^ and Khosravi et al.^16^ demonstrated that deep convolutional neural networks could accurately classify NSCLC histopathology images into subtypes (adenocarcinoma and squamous cell carcinoma) and even predict genetic mutations. Furthermore, Ardila et al.^17^ proposed an end-to-end 3D deep learning model for lung cancer screening, achieving performance comparable to expert radiologists. Despite these breakthroughs, the majority of existing CAD systems treat lesion localization, subtyping, and TNM staging as independent, isolated tasks. Our work distinguishes itself by formulating a unified, fine-grained object detection framework capable of simultaneously outputting the lesion bounding box, radiologic subtype, specific anatomical location, and clinical TNM stage.

### Object detection and YOLO architectures

Modern object detection frameworks are generally categorized into two-stage detectors (e.g., Faster R-CNN^18^) and one-stage detectors (e.g., YOLO series). The YOLO (You Only Look Once) family^19^, characterized by its unified network structure, has become the dominant paradigm for real-time CAD applications due to its optimal balance between detection accuracy and inference speed.

Recent advancements in the YOLO architecture, such as YOLOv7^20^, YOLOv9^21^, YOLOv10 and YOLOv11, have further optimized the backbone and eliminated the need for non-maximum suppression (NMS), enabling true end-to-end detection. However, standard YOLO models are primarily designed for natural images with prominent object features. When directly applied to medical imaging, they often struggle with the subtle inter-class differences of tumors and complex anatomical backgrounds, challenges that traditionally require specialized optimization strategies like Focal Loss^22^. Therefore, this paper introduces targeted structural modifications to the latest YOLO architecture, making it highly suitable for the complex 7-class fine-grained lung cancer detection task.

### Multi-scale perception and attention mechanisms

Effectively extracting multi-scale features is critical in medical image analysis, as lung tumors exhibit extreme variations in size across different clinical stages. Traditional methods alleviate this issue by utilizing Feature Pyramid Networks (FPN)^6^ to fuse hierarchical features. Recently, large-kernel convolutions have seen a resurgence. Ding et al.^8^ proposed UniRepLKNet, demonstrating that large-kernel and dilated convolutions can significantly expand the receptive field, mimicking the global perception of Vision Transformers (ViTs)^23^ while maintaining the efficiency of CNNs via structural reparameterization.

In addition to scale variance, suppressing background noise is equally important. Attention mechanisms, such as the Squeeze-and-Excitation (SE) network^24^, the Convolutional Block Attention Module (CBAM)^11^, and attention-gated networks specifically designed for medical imaging^25^, have been widely adopted to help models focus on salient regions. Building upon the Pointwise Spatial Attention (PSA) module^12^, we design an Explicit Positional Gating Optimization (EPGO) module. Unlike static attention operators, EPGO dynamically calculates the keys for self-attention, enabling the network to adaptively emphasize discriminative radiologic textures and effectively differentiate between highly similar NSCLC subtypes.

### Feature upsampling and fusion

In the feature fusion stage (Neck), upsampling operators play a vital role in recovering spatial resolution from deep, low-resolution feature maps. Most existing object detectors employ nearest-neighbor or bilinear interpolation for upsampling. However, these fixed, content-agnostic operators often lead to the loss of fine-grained spatial details, which is particularly detrimental in medical imaging where tumor boundaries are inherently blurred and irregular, a geometric challenge often addressed by complex operations like deformable convolutions^26^. To overcome this limitation, Liu et al.^13^ proposed DySample, a lightweight dynamic upsampling technique that learns point sampling offsets directly from the input features. By integrating DySample into our feature fusion network, our model achieves content-aware spatial alignment, maximally preserving the edge details of lung lesions and ultimately enhancing the precision of bounding box localization.

## Methodology

### Overall architecture of the proposed network

**Fig. 1:**
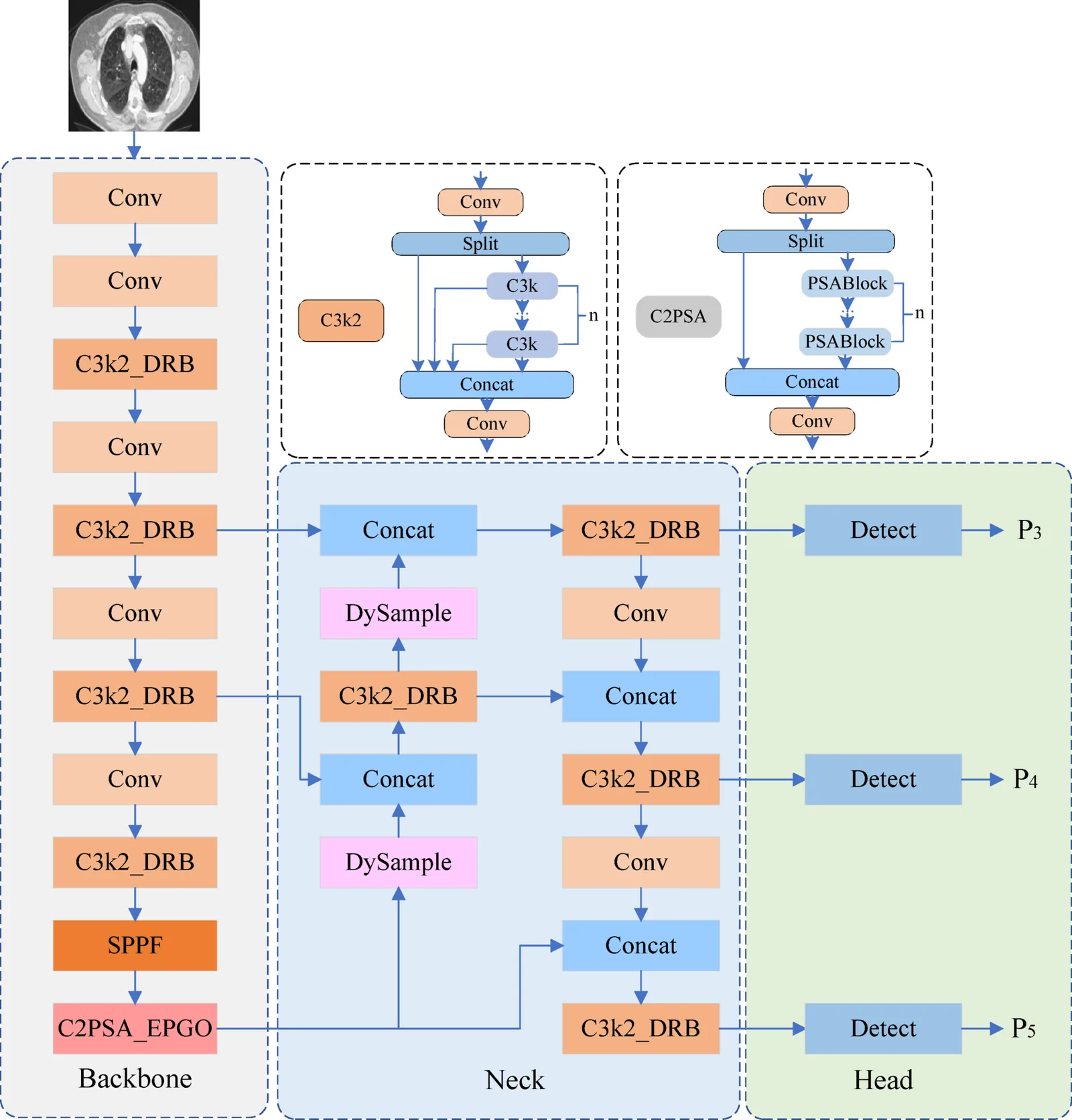
The overall architecture of the proposed fine-grained lung cancer object detection network.

To tackle the complex 7-class lung cancer detection task, which involves simultaneous lesion localization, fine-grained radiologic subtyping, and TNM clinical staging, we propose an enhanced end-to-end object detection network built upon the state-of-the-art YOLO11n architecture, as illustrated in Fig. 1. The overall network consists of three main components: the backbone, the neck, and the detection head. Because standard convolutional blocks in the backbone are inadequate for capturing the massive scale variations of lung tumors across different clinical stages, we introduce the Dilated Reparameterization Block (DRB) to construct the novel C3k2_DRB module. This structure leverages multi-scale dilated convolutions to effectively expand the receptive field without imposing a heavy computational burden during inference. Furthermore, in the deepest layer of the backbone, we integrate the Explicit Positional Gating Optimization (EPGO) attention mechanism within the C2PSA_EPGO module, allowing the network to adaptively focus on the most discriminative radiologic textures and mitigate interference from complex anatomical backgrounds. Moving to the neck feature fusion stage, we replace traditional nearest-neighbor interpolation with the DySample module, a dynamic upsampling strategy that enables content-aware spatial alignment to strictly preserve the highly irregular and blurred boundary details of lung lesions. Finally, these refined, fused multi-scale features are fed into the detection head to accurately output the precise bounding boxes and their corresponding fine-grained clinical categories.

### C3k2-DRB module for multi-scale feature extraction

**Fig. 2:**
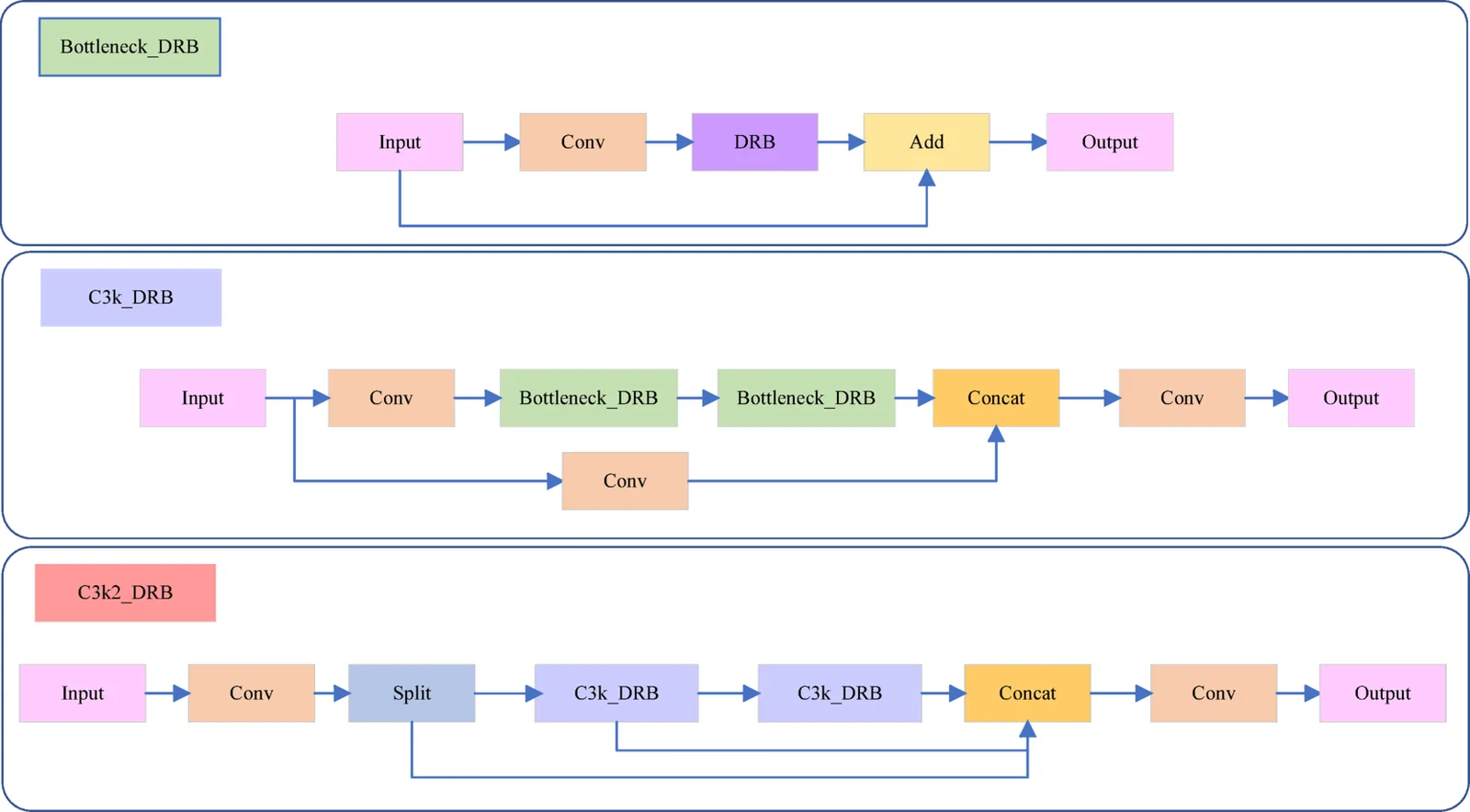
C3k2_DRB module structure diagram.

In medical image analysis, lung tumors across different TNM stages exhibit massive variations in scale. Early-stage cancer cells may manifest as microscopic nodules, whereas advanced-stage large cell carcinomas often occupy a significant portion of the anatomical structure. Standard convolutions with fixed, small receptive fields (e.g., *3 × 3*) struggle to capture such broad spatial context. To address this, we construct a novel hierarchical feature extraction module named C3k2_DRB, as illustrated in Fig. 2. The design of this module is highly modular and consists of three nested levels: the Bottleneck_DRB, the C3k_DRB, and the overarching C3k2_DRB structure.

#### Bottleneck_DRB with structural reparameterization

As shown in the top dashed box of Fig. 2, the fundamental building block is the Bottleneck_DRB. Traditional bottleneck structures use standard spatial convolutions, which have a limited perceptual range. We replace the primary spatial convolution with the proposed Dilated Reparameterization Block (DRB). For an input feature map $X \in {\mathbb{R}}^{C \times H \times W}$, the DRB leverages a large-kernel primary branch alongside multiple parallel dilated convolutional branches during the training phase. The output $Y_{train}$ is formulated as:

1 $$Y_{train} = {\mathrm{BN}}_{{{\mathrm{origin}}}} (X*W_{{{\mathrm{origin}}}} ) + \sum\limits_{i = 1}^{N} {{\mathrm{BN}}_{i} } (X*W_{{k_{i} ,r_{i} }} )$$

where * denotes the convolution operation, ${\mathrm{BN}}$ is the Batch Normalization layer, $W_{{{\mathrm{origin}}}}$ is the weight of the primary large kernel (e.g., *7 × 7*), and $W_{{k_{i} ,r_{i} }}$ represents the weights of the *i*-th parallel branch with kernel size $k_{i}$ and dilation rate $r_{i}$.

To ensure extremely fast inference speed for clinical deployment without sacrificing multi-scale representation, we utilize a structural reparameterization mechanism inspired by RepVGG^27^. Since the convolution and BN are linear transformations, their parameters (BN scaling factor *γ*, shift *β*, mean *μ*, and variance $\sigma^{2}$) can be fused with the original convolutional weights *W* into an equivalent convolution weight $W_{eq}$ and bias $b_{eq}$ to eliminate BN overhead during inference. The fusion is formulated as:

2 $$W_{eq} = \frac{\gamma }{{\sqrt {\sigma^{2} + \varepsilon } }}W$$

3 $$b_{eq} = - \frac{\mu \gamma }{{\sqrt {\sigma^{2} + \varepsilon } }} + \beta$$

where *\varepsilon* is a small constant added for numerical stability. Subsequently, the dilated kernels are transformed into a dense equivalent kernel via a spatial zero-padding operator ${\mathcal{P}}( \cdot ,r_{i} )$. The final deployment parameters $(W_{deploy} ,B_{deploy} )$ are derived by summing all branches:

4 $$W_{deploy} = W_{{{\mathrm{origin}}}}^{\prime } + \sum\limits_{i = 1}^{N} {\mathcal{P}} (W_{{k_{i} ,r_{i} }}^{\prime } ,r_{i} )$$

5 $$B_{deploy} = B_{{{\mathrm{origin}}}}^{\prime } + \sum\limits_{i = 1}^{N} {B_{{k_{i} ,r_{i} }}^{\prime } }$$

Thus, during the inference phase, the Bottleneck_DRB executes a single, highly efficient operation: $Y_{deploy} = X*W_{deploy} + B_{deploy} + X$, where the final *+ X* represents the residual shortcut preserving gradient flow.

#### C3k_DRB for feature reuse

Building upon the Bottleneck_DRB, we design the C3k_DRB module, depicted in the middle section of Fig. 2. Inspired by the Cross Stage Partial (CSP) network design^28^, the input features $F_{in}$ are processed through an initial *1 × 1* convolution to adjust the channel dimension to $C^{\prime }$. The feature map is then split into two distinct gradient paths: a main processing path and a residual shortcut. If we denote the sequence of *n* stacked Bottleneck_DRB operations as ${\mathcal{F}}_{{{\mathrm{DRB}}}}^{n} ( \cdot )$, the bifurcated feature processing can be modeled as:

6 $$F_{{{\mathrm{main}}}} = {\mathcal{F}}_{{{\mathrm{DRB}}}}^{n} (F_{in}^{(1)} )$$

7 $$F_{{{\mathrm{res}}}} = {\mathrm{Conv}}_{1 \times 1} (F_{in}^{(2)} )$$

Finally, the features from both paths are concatenated along the channel dimension and fused via a projection layer:

8 $$Y_{{{\mathrm{C3k}}}} = {\mathrm{Conv}}_{{{\mathrm{proj}}}} ([F_{{{\mathrm{main}}}} ,F_{{{\mathrm{res}}}} ])$$

where $[ \cdot , \cdot ]$ denotes the concatenation operation. This design significantly enhances feature reuse, reduces computational redundancy, and ensures stable gradient backpropagation during the training of deep architectures.

#### C3k2_DRB architecture

At the macro level, the C3k2_DRB module acts as the primary feature extraction stage in our improved YOLO backbone. Drawing inspiration from the ELAN (Efficient Layer Aggregation Network) architecture^29^, the input $Z_{in}$ undergoes a base convolution and a split operation to form initial features $Z_{0}$ and $Z_{c}$. The feature $Z_{0}$ is then sequentially routed through *M* sequential C3k_DRB blocks. The intermediate output of the *j*-th block is given by:

9 $$Z_{j} = {\mathrm{C3k}}\_{\mathrm{DRB}}_{{\mathrm{j}}} ({\mathrm{Z}}_{{\text{j - 1}}} ),\quad {\mathrm{j}} \in \{ {1},{2},...,{\mathrm{M}}\}$$

Crucially, intermediate outputs from these blocks are dynamically routed and aggregated. The final multi-scale representation $Z_{out}$ is generated by concatenating the shortcut feature $Z_{c}$ with the outputs of all intermediate blocks:

10 $$Z_{out} = {\mathrm{Conv}}_{{{\mathrm{final}}}} ([Z_{c} ,Z_{1} ,Z_{2} ,...,Z_{M} ])$$

This rich gradient aggregation strategy ensures that semantic information at multiple scales, ranging from local cellular abnormalities to global lobar structures, is optimally integrated.

### Explicit positional gating optimization (EPGO) attention

**Fig. 3:**
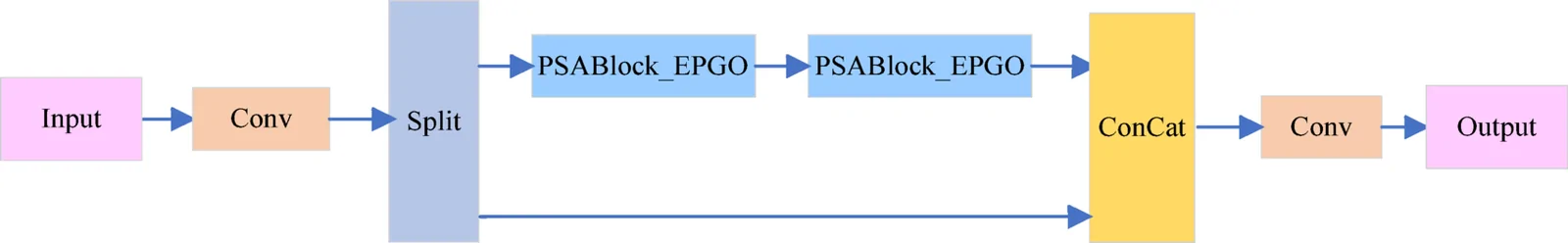
C2PSA_EPGO module structure diagram.

While the C3k2_DRB module efficiently extracts multi-scale spatial features, lung cancer medical images inherently contain vast areas of irrelevant anatomical backgrounds, such as ribs, healthy lung parenchyma, and airways. Standard self-attention mechanisms treat all spatial tokens equally, which not only introduces quadratic computational complexity but also risks amplifying background noise, thereby diminishing the subtle inter-class differences between NSCLC subtypes. To address this, we incorporate an enhanced Explicit Positional Gating Optimization (EPGO) attention mechanism. Built upon the Pointwise Spatial Attention (PSA) framework^12^, the EPGO module utilizes a lightweight gating network to predict image-specific attention thresholds^25^, enabling the model to adaptively suppress background noise while focusing on discriminative lesion textures. This mechanism is integrated into the C2PSA_EPGO module, as illustrated in Fig. 3.

#### Macro-architecture of C2PSA_EPGO

As depicted in Fig. 3, the C2PSA_EPGO module adopts a Cross Stage Partial (CSP) architecture to optimize gradient flow. An input feature map $X \in {\mathbb{R}}^{C \times H \times W}$ is first projected and split into two parallel branches. The main branch processes the features through *N*(*N = 2*)sequential PSABlock_EPGO modules, which compute high-level, globally attended representations. The secondary branch serves as a structural identity shortcut. Finally, the outputs from both branches are concatenated along the channel dimension and fused via a convolution. This bifurcated design prevents the vanishing gradient problem in deep attention networks while maintaining high feature diversity.

#### Dynamic gating mechanism (EPGO)

The core innovation within the PSABlock_EPGO is the dynamically gated self-attention. Traditional sparse attention methods rely on a fixed hyperparameter to drop tokens, which is sub-optimal given the dramatic size variations of lung tumors. Our EPGO module actively learns to predict the optimal proportion of tokens to attend to, based directly on the spatial content of the image.

Given a flattened input sequence $X \in {\mathbb{R}}^{N \times C}$ (where *N = H × W*), we first compute the queries *Q*, keys *K*, and values *V* through linear projections. To determine the dynamic attention threshold, we design a lightweight gating network ${\mathcal{G}}( \cdot )$ composed of stacked *1 × 1* convolutions, a ReLU activation, and a Sigmoid function. The gating network predicts a continuous spatial probability map, which is then globally averaged to compute a dynamic retention ratio *γ*:

11 $$\gamma = \frac{1}{N}\sum\limits_{i = 1}^{N} \sigma \left( {{\mathrm{Conv}}_{1 \times 1} ({\mathrm{ReLU}}({\mathrm{Conv}}_{1 \times 1} (X_{i} )))} \right)$$

where $\gamma \in (0,1)$. This ratio represents the image-specific proportion of relevant foreground (tumor) pixels. The dynamic number of key tokens $K_{dynamic}$ to be retained is thus calculated as:

12 $$K_{dynamic} = \left\lfloor {N \cdot \gamma } \right\rfloor$$

This content-aware calculation ensures that images with extensive tumor infiltration preserve more tokens, whereas images with tiny micro-nodules aggressively filter out the dominant healthy background.

#### Top-K sparse attention and positional encoding

With $K_{dynamic}$ determined, we compute the raw attention score matrix $A = (QK^{T} )/\sqrt d$, where *d* is the scaling factor. Rather than computing the standard dense softmax, we apply a row-wise Top-K selection to construct a dynamic binary mask ${\mathcal{M}} \in {\mathbb{R}}^{N \times N}$. For the *i*-th row, the mask is defined as:

13 $${\mathcal{M}}_{i,j} = \left\{ {\begin{array}{*{20}l} {1,} \hfill & {{\text{if }}A_{i,j} {\text{ is in the top }}K_{dynamic} {\text{ values of }}A_{i} } \hfill \\ {0,} \hfill & {{\mathrm{otherwise}}} \hfill \\ \end{array} } \right.$$

The attention matrix is then masked by replacing the unselected connections with negative infinity before applying the Softmax activation. Finally, to compensate for the loss of local spatial inductive bias inherent in self-attention, we introduce an explicit convolutional positional encoding function ${\mathcal{P}\mathcal{E}}( \cdot )$ to the values *V*. The final output of the EPGO attention $Y_{EPGO}$ is computed as:

14 $$Y_{EPGO} = {\mathrm{Softmax}}\left( {A \odot {\mathcal{M}} + (1 - {\mathcal{M}})( - \infty )} \right)V + {\mathcal{P}\mathcal{E}}(V)$$

By seamlessly integrating dynamic token gating with explicit positional encoding, the EPGO module aggressively suppresses background anatomical noise while sharply capturing the highly discriminative, long-range radiologic dependencies required for accurate NSCLC subtyping and TNM staging.

### Dynamic feature upsampling (DySample)

In the feature fusion stage (Neck), recovering high-resolution spatial details from deep, low-resolution feature maps is critical for precise bounding box regression. Standard object detectors typically employ nearest-neighbor or bilinear interpolation for upsampling. However, these fixed, content-agnostic operators apply uniform spatial mappings across the entire image. Given that lung tumors often possess highly irregular, blurred, and complex boundaries (especially for advanced NSCLC subtypes invading adjacent tissues), standard upsampling inevitably leads to semantic misalignment and the loss of fine-grained morphological contours.

**Fig. 4:**
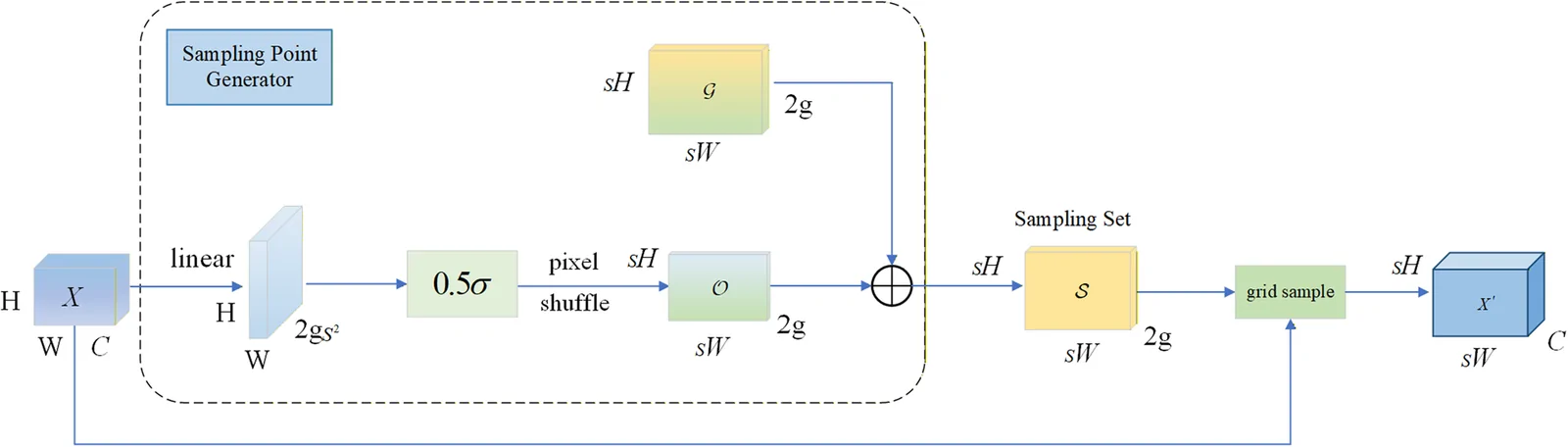
Dynamic feature upsampling.

To overcome this limitation, we introduce the DySample module, a lightweight and dynamic upsampling operator, as illustrated in Fig. 4. Instead of relying on dynamic convolutions which introduce significant computational overhead, DySample achieves content-aware upsampling from the perspective of point sampling.

Given an input feature map $X \in {\mathbb{R}}^{C \times H \times W}$, our goal is to upsample it to $X^{\prime } \in {\mathbb{R}}^{C \times sH \times sW}$, where *s* is the upsampling scale factor. DySample achieves this by generating a dynamic Sampling Set ${\mathcal{S}}$.

As shown in the dashed box of Fig. 4, DySample first employs a linear projection layer (implemented as a *1 × 1* convolution) to generate the raw spatial offsets. To enhance the flexibility of the sampling locations, a dynamic scope mechanism is introduced. The generated features are passed through a Sigmoid activation (*σ*) and scaled by a factor of 0.5. Subsequently, a pixel_shuffle operation reshapes the tensor to match the target high-resolution spatial dimensions. The generated continuous spatial offset ${\mathcal{O}} \in {\mathbb{R}}^{2g \times sH \times sW}$ (where *g* is the number of groups) can be mathematically formulated as:

15 $${\mathcal{O}} = {\mathrm{PixelShuffle}}\left( {{\mathrm{Conv}}_{1 \times 1} (X) \odot 0.5\sigma ({\mathrm{Conv}}_{scope} (X))} \right)$$

Alternatively, in a simplified deployment without the scope gating, the offset is computed as ${\mathcal{O}} = {\mathrm{PixelShuffle}}({\mathrm{Conv}}_{1 \times 1} (X)) \times 0.25$.

Let ${\mathcal{G}} \in {\mathbb{R}}^{2g \times sH \times sW}$ represent the standard, uniform regular sampling grid (i.e., the original normalized coordinates). The dynamic Sampling Set ${\mathcal{S}}$ is constructed by element-wise addition of the learned offsets ${\mathcal{O}}$ to the initial regular grid ${\mathcal{G}}$:

16 $${\mathcal{S}} = {\mathcal{G}} + {\mathcal{O}}$$

Finally, using the dynamic coordinates defined in ${\mathcal{S}}$, the upsampled feature map $X^{\prime }$ is obtained by sampling the original input *X* via the bilinear grid_sample function:

17 $$X^{\prime } = {\mathrm{grid}}\_{\mathrm{sample}}(X,{\mathcal{S}})$$

By learning dynamic point sampling offsets ${\mathcal{O}}$, the DySample module adaptively adjusts its sampling locations based on the semantic content of the feature map. For pixels located at the boundaries of a lung lesion, the offsets intuitively “pull” features from the actual tumor region rather than the surrounding healthy lung parenchyma. This dynamic alignment strictly preserves the sharp boundaries and morphological details of lung cancer lesions, ultimately contributing to higher localization precision and more accurate TNM clinical staging.

## Experiments

### Dataset

The primary data source for this study is the publicly available Chest CT-Scan images Dataset (accessed via Kaggle: https://www.kaggle.com/datasets/mohamedhanyyy/chest-ctscan-images), which consists of a comprehensively curated collection of 1886 medical images. To rigorously evaluate the proposed model’s capability in fine-grained radiologic feature classification and clinical staging, we utilized the pre-existing labels provided within this Kaggle dataset, which meticulously annotate the images into 7 distinct categories. These categories represent a hierarchical clinical diagnostic progression: beginning with baseline tissue states (normal and cancer-cells), advancing to the radiologic presentations of the three major non-small cell lung cancer (NSCLC) subtypes (adenocarcinoma, large-cell-carcinoma, and squamous-cell-carcinoma), and culminating in highly complex, stage-specific classifications that integrate the radiologic subtype, anatomical location, and specific TNM clinical stage (i.e., adenocarcinoma_left-lower-lobe_T2_N0_M0_Ib, large-cell-carcinoma_left-hilum_T2_N2_M0_IIIa, and squamous-cell-carcinoma_left-hilum_T1_N2_M0_IIIa).

It is critical to acknowledge that this open-source dataset lacks rigorous, peer-reviewed clinical validation and does not represent a definitive clinical gold standard. From a strict clinical perspective, reliable biomarkers for fine-grained histological subtyping (e.g., differentiating adenocarcinoma from squamous cell carcinoma) and comprehensive TNM staging—particularly Lymph Node (N) and Metastasis (M) statuses—cannot be definitively inferred from a single 2D CT slice. Therefore, it is highly probable that the dataset contains inherent annotation biases or device-related artifacts. Consequently, the 7-class detection task defined in this study is utilized strictly as a highly challenging computationally algorithmic benchmark, rather than a proposed clinical diagnostic pathway. It serves to computationally evaluate the proposed network’s capacity to extract subtle features and differentiate highly overlapping visual classes in a complex, multi-scale scenario. It is highly suitable for evaluating the feature extraction capabilities and the proof-of-concept utility of the proposed object detection network in complex, fine-grained visual tasks.

To ensure that the model has sufficient data to learn complex feature representations while providing a reliable benchmark for evaluation, the dataset was strictly partitioned with specific target quotas. Following a standard 8:1:1 splitting protocol, the 1886 images were distributed as follows: 1509 images were allocated to the training set to optimize the network weights, 189 images to the validation set for hyperparameter tuning and model selection, and the remaining 188 images to the testing set for the final unbiased performance assessment. This rigorous partitioning strategy guarantees that the reported metrics accurately reflect the proposed model’s generalization capability and robustness on unseen clinical data. The detailed distribution of sample quantities across the 7 fine-grained classes is presented in Table 1.

**Table 1:** Detailed dataset partition across different fine-grained classes.

Class Name	Train	Val	Test	Total
normal	136	16	17	169
adenocarcinoma	164	13	23	200
adenocarcinoma_left-lower-lobe_T2_N0_M0_Ib	542	74	68	684
large-cell-carcinoma	47	7	6	60
large-cell-carcinoma_left-hilum_T2_N2_M0_IIIa	174	20	18	212
squamous-cell-carcinoma	374	49	48	471
squamous-cell-carcinoma_left-hilum_T1_N2_M0_IIIa	70	12	8	90
Total annotated instances	1507	191	188	1886

### Evaluation metrics

To quantitatively assess the detection performance, we employ standard object detection metrics, including Precision (P), Recall (R), and mean Average Precision (mAP). The *mAP* is calculated across all 7 classes and is the primary metric for comprehensive evaluation, particularly mAP0.5 and mAP0.5:0.95. The formulas for Precision and Recall are defined as:

18 $$P = \frac{TP}{{TP + FP}}$$

19 $$R = \frac{TP}{{TP + FN}}$$

where *TP*, *FP*, and *FN* denote the number of true positives, false positives, and false negatives, respectively. Furthermore, to evaluate the clinical deployment potential and computational efficiency of the models, we also report the number of parameters (Params), floating-point operations per second (GFLOPs), and inference speed in Frames Per Second (FPS).

### Implementation details

All experiments were conducted on a high-performance deep learning workstation to ensure training efficiency and reproducibility. The hardware environment was equipped with a single NVIDIA GeForce RTX 4090 GPU with 24 GB of VRAM, supported by an AMD EPYC 7T83 64-Core Processor utilizing 22 virtual CPUs (vCPUs). The software stack was built on the Ubuntu 20.04 operating system, employing Python 3.8 and the PyTorch 2.0.0 deep learning framework. Furthermore, CUDA 11.8 was utilized to enable efficient GPU acceleration during the intensive tensor computations required by the multi-scale structural reparameterization and dynamic attention mechanisms.

During the training phase, the input medical images were uniformly resized to *640 × 640* pixels to balance computational efficiency and high-resolution feature retention. The proposed network was optimized using the Stochastic Gradient Descent (SGD) optimizer. The initial learning rate was set to 0.01, accompanied by a momentum of 0.937 and a weight decay of 0.0005 to prevent overfitting. The entire training process spanned 200 epochs with a batch size of 64. To further enhance the robustness of the model against varying lung tumor morphologies and complex anatomical backgrounds, standard data augmentation strategies, including mosaic, random scaling, and horizontal flipping, were applied within the training pipeline.

Crucially, these strategies also served as a primary countermeasure against the inherent class imbalance present in our fine-grained dataset. Specifically, Mosaic augmentation combines multiple images into a single training batch, structurally increasing the exposure and contextual diversity of minority class instances (e.g., large-cell-carcinoma). Furthermore, to address the long-tail distribution algorithmically, the model’s loss function incorporates an automatic class-weighting mechanism during training. This strategy scales the classification loss penalties inversely proportional to the class frequencies, ensuring that the network remains highly sensitive to underrepresented radiologic subtypes and prevents learning bias toward the majority classes.

## Experimental results and analysis

### Comparison with state-of-the-art methods

To evaluate the effectiveness of the proposed model in lung cancer detection, we benchmarked it against several state-of-the-art (SOTA) object detection models on our dataset. The compared models include YOLOv8n^30^, YOLOv10s, YOLOv12n^31^, YOLOv12s, and the baseline YOLOv11n^32^. The evaluation focuses on balancing model complexity and detection accuracy, utilizing parameter count (Params), computational complexity (GFLOPs), Precision, and mean Average Precision (mAP@0.5) as the primary metrics. The quantitative results are summarized in Table 2.

**Table 2:** Performance comparison of different object detection models on the lung cancer dataset.

Model	Params (M)	GFLOPs	Precision (%)	mAP@0.5 (%)
YOLOv8n	3.01	8.1	95.8	98.9
YOLOv10s	8.04	24.5	93.4	98.5
YOLOv12n	2.51	5.8	93.4	97.4
YOLOv12s	9.08	19.3	97.1	98.8
YOLOv11n (baseline)	2.58	6.3	95.2	98.1
Ours	2.46	6.3	97.6	99.2

As shown in Table 2, the proposed model achieves an optimal balance between detection accuracy and inference efficiency. It attains the highest Precision (97.6%) and mAP@0.5 (99.2%) among all evaluated models. In medical image analysis, this high Precision is crucial for maintaining a critically low false-positive rate to avoid unnecessary patient anxiety. Compared to the YOLOv11n baseline, our model improves Precision by 2.4% and mAP@0.5 by 1.1% while reducing parameters (from 2.58 to 2.46 M) and maintaining identical computational complexity (6.3 GFLOPs). Furthermore, it demonstrates a comprehensive advantage over other state-of-the-art models. It significantly outperforms recent Nano-scale models like YOLOv12n (+ 4.2% Precision, + 1.8% mAP@0.5) with fewer parameters. Remarkably, it even surpasses larger Small-scale models (YOLOv10s, YOLOv12s) in all accuracy metrics while utilizing less than one-third of their hardware resources. In conclusion, by breaking the baseline’s performance bottleneck without adding computational burden, the proposed model proves highly feasible for real-time, high-precision deployment on computing-constrained clinical edge devices.

To further comprehensively evaluate the localization precision, diagnostic reliability, and real-time applicability of the models, we present the Recall, mAP@0.5:0.95, and inference speed (FPS) in Table 3.

**Table 3:** Comparison of localization precision, recall, and real-time processing speed.

Model	Recall	mAP@0.5:0.95	Inference (ms)	Total (ms)	FPS
YOLOv8n	94.1	98.9	0.8	3.4	294
YOLOv10s	95.2	98.5	1.7	3.8	263
YOLOv12n	94.3	89.3	0.6	2.1	476
YOLOv12s	92.4	98.8	1.3	2.8	357
YOLOv11n	93.1	98.1	0.4	1.9	526
Ours	93.2	99.2	3.6	5.0	200

To further evaluate the localization precision and real-time applicability of the models, we present the Recall, mAP@0.5:0.95, and inference speed in Table 3. Notably, a significant performance gap is observed in YOLOv12n, which drops from 97.4 to 89.3%. This discrepancy highlights a fundamental bottleneck in standard architectures: relying on fixed, content-agnostic upsampling operators (such as nearest-neighbor interpolation) leads to semantic misalignment and the loss of fine-grained spatial details, especially for lung tumors with blurred and irregular boundaries. Consequently, these baseline models tend to generate slightly oversized or ‘loose’ bounding boxes that easily pass the 50% IoU threshold but fail under stricter criteria. In contrast, the proposed model avoids this penalty and achieves the highest mAP@0.5:0.95 of 99.2%, outperforming both the baseline YOLOv11n and the larger YOLOv12s. This proves that the introduced modules, particularly the DySample dynamic upsampling strategy, successfully achieve content-aware spatial alignment to accurately preserve lesion contours and tighten bounding box localization. In terms of processing speed, the proposed model takes 5.0 ms per image. Although the computational overhead of the C2PSA_EPGO mechanism and dynamic sampling reduces the speed compared to the baseline YOLOv11n, a frame rate of 200 FPS is still sufficient for standard real-time clinical deployment. This indicates a reasonable trade-off between localization precision and inference speed.

**Fig. 5:**
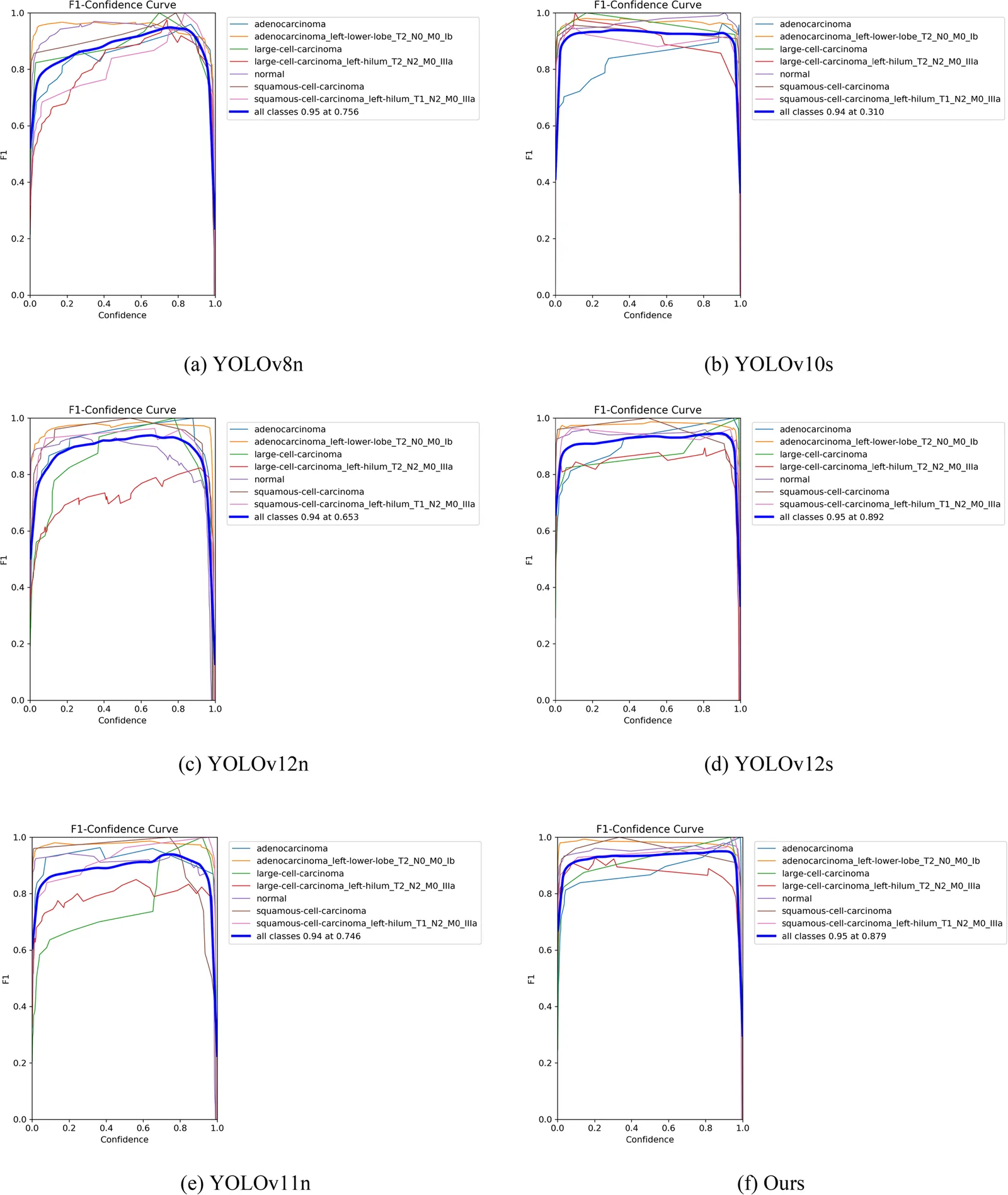
Comparison of F1-Confidence curves among different object detection models on the lung cancer dataset.

The F1-Confidence curves for the evaluated models are presented in Fig. 5. The F1 score, representing the harmonic mean of precision and recall, evaluates a model’s comprehensive performance across varying confidence thresholds. A higher, broader curve indicates superior robustness. As illustrated, the proposed model (Ours, Fig. 5f) achieves an outstanding peak F1 score of 0.95 at a confidence threshold of 0.879. Remarkably, this peak matches that of the significantly heavier YOLOv12s model. Furthermore, it surpasses the baseline YOLOv11n, as well as other lightweight variants like YOLOv10s and YOLOv12n. Beyond reaching a higher peak, the proposed model exhibits remarkable stability. The all-class curve maintains a consistently high, flat plateau, with the F1 score remaining above 0.90 across an extremely wide confidence range from approximately 0.1 to 0.9. In contrast, the baseline YOLOv11n (Fig. 5e) demonstrates a steep performance drop at lower confidence levels. This broad plateau proves our model is highly robust and insensitive to threshold variations, providing a crucial advantage for reliable clinical deployment. Finally, comparing the individual class curves (thin lines) reveals a significant improvement in detecting traditionally difficult classes. For instance, while the baseline model struggles with large-cell-carcinoma at lower confidences, our model substantially elevates this specific curve. Consequently, all individual class curves converge much more tightly. This demonstrates that our architectural improvements successfully enhance feature extraction for challenging lesions, ensuring a more balanced and clinically viable detection capability across diverse lung cancer subtypes.

**Fig. 6:**
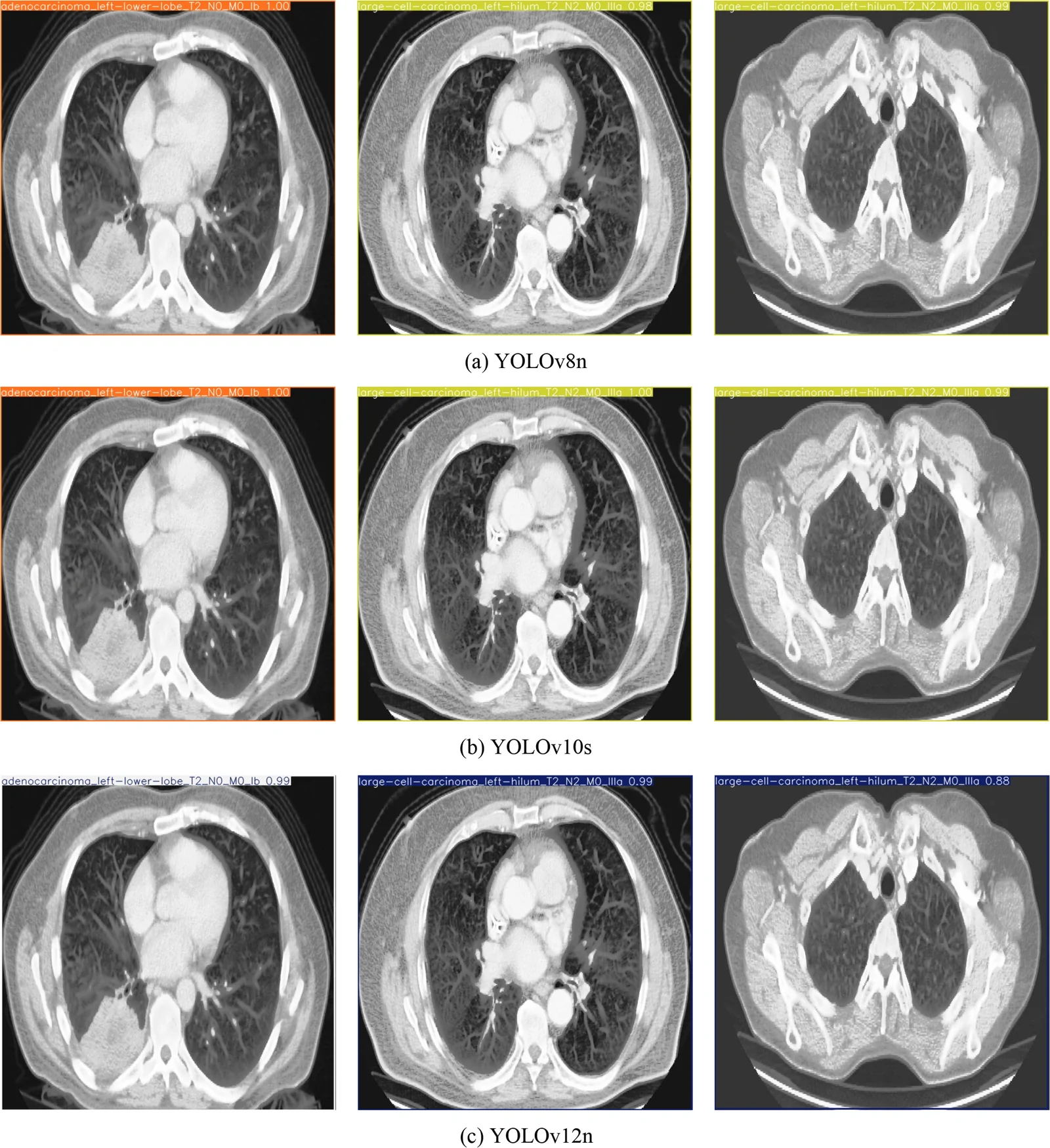

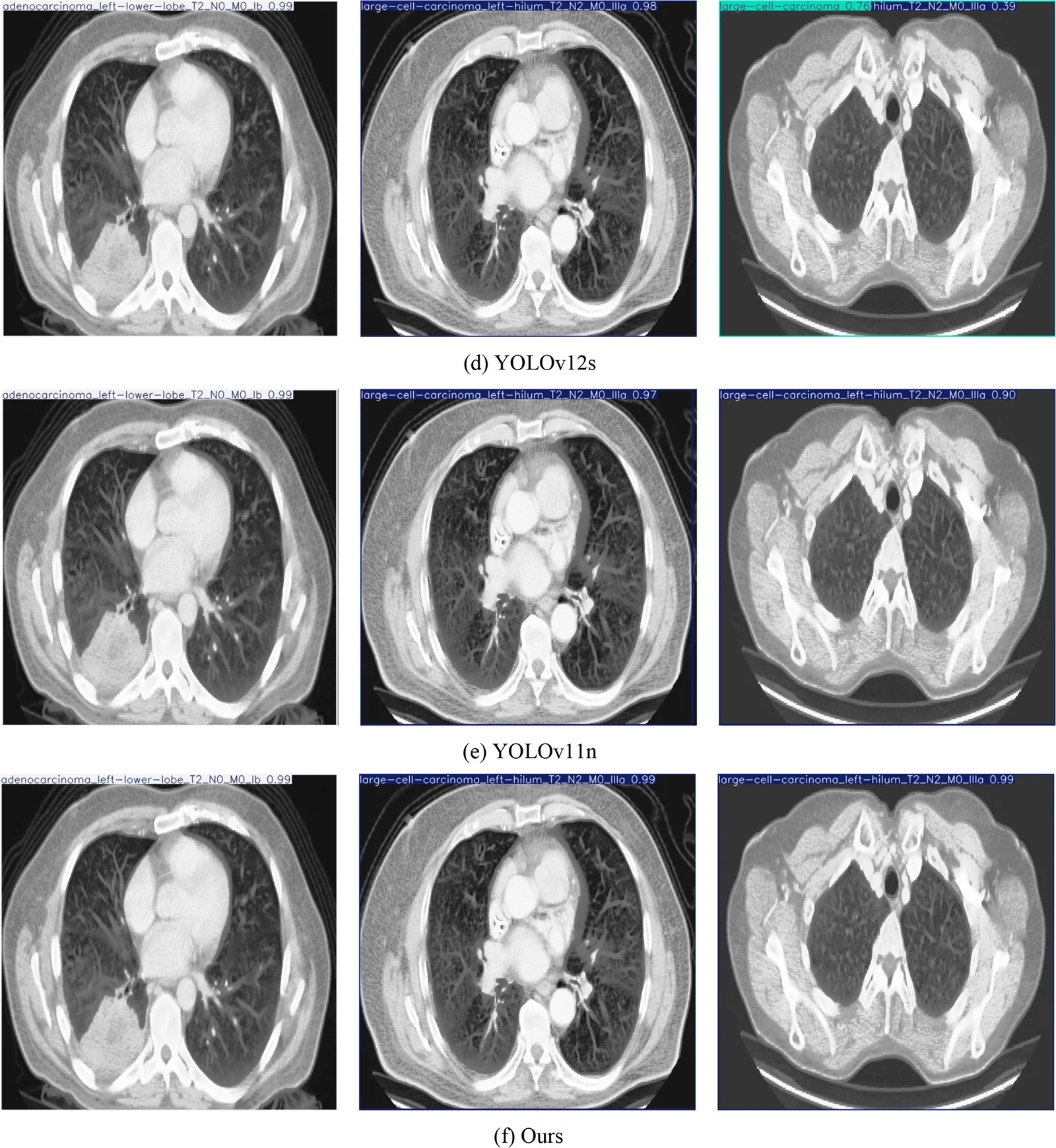
Qualitative comparison of detection results between the proposed model and mainstream baselines on complex lung cancer samples.

To thoroughly evaluate the proposed model’s practical utility in complex clinical scenarios, we visualized and compared its detection results against several mainstream baselines, including YOLOv10s, YOLOv12s, and the baseline YOLO11n, using highly challenging non-small cell lung cancer (NSCLC) samples. As illustrated in the visual comparisons in Fig. 6, our proposed method demonstrates clear, distinct improvements across three critical aspects: bounding box tightness, confidence score maximization, and robust background noise suppression. Specifically, when evaluating the adenocarcinoma_left-lower-lobe_T2_N0_M0_Ib case (left column), the baseline models consistently generate oversized bounding boxes that erroneously encompass surrounding healthy lung parenchyma. In sharp contrast, our proposed model accurately produces a much tighter bounding box that closely conforms to the highly irregular contours of the tumor. Furthermore, for the substantial large-cell-carcinoma_left-hilum_T2_N2_M0_IIIa case (middle column), our method achieves an exceptional confidence score of 0.99, outperforming the baseline YOLO11n’s score of 0.97. This highlights the network’s enhanced ability to capture and process broad spatial contexts for larger lesions. Moreover, in a highly complex large-cell-carcinoma case (right column) where the baseline models severely struggle, producing either redundant, overlapping boxes or significantly lower confidence scores, our network successfully outputs a single, precise bounding box with a high confidence of 0.99. In summary, these qualitative visualizations align with our quantitative results, confirming the model’s capacity for accurate fine-grained sub-category identification and highly reliable tumor localization, which is essential for advanced clinical surgical planning.

**Fig. 7:**
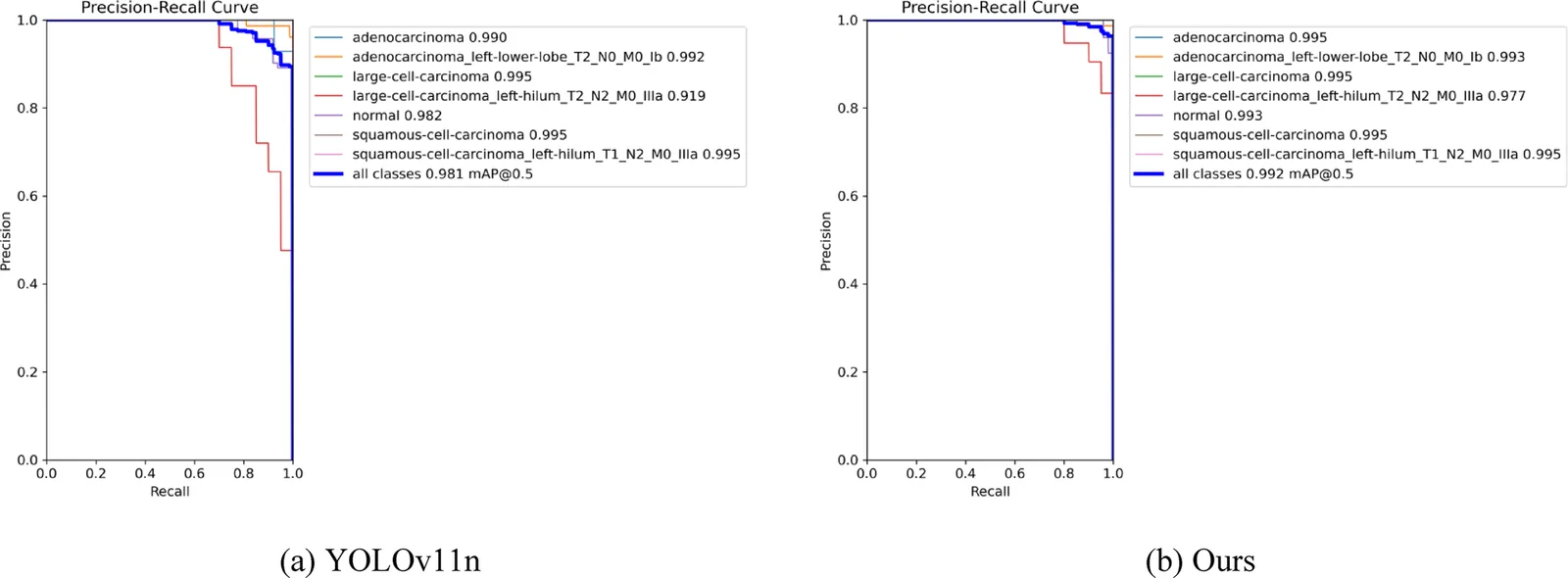
Comparison of precision-recall (P-R) curves between YOLOv11n and the proposed model (Ours).

Figure 7 illustrates the comparison of the Precision-Recall (P-R) curves between YOLOv11n and the proposed model (Ours), indicating that the proposed model achieves performance improvements both overall and across the majority of categories. Specifically, the overall mean average precision (mAP@0.5) of the proposed model increases from 0.981 in the baseline model to 0.992, with the curve shifting closer to the top-right corner, demonstrating superior comprehensive detection performance. Regarding subcategories, the proposed model yields a noticeable improvement in a specific stage of large cell carcinoma (large-cell-carcinoma_left-hilum_T2_N2_M0_IIIa), which underperformed in the baseline model, increasing its AP value from 0.919 to 0.977. Simultaneously, for conventional categories such as normal tissue (from 0.982 to 0.993), adenocarcinoma (from 0.990 to 0.995), and its specific subcategory (from 0.992 to 0.993), the precision of the proposed model exhibits a marginal yet stable increase. Furthermore, for categories such as large cell carcinoma and squamous cell carcinoma, both models maintain an AP value of 0.995, demonstrating relatively consistent and stable performance.

### Ablation study

To rigorously validate the individual contributions and the synergistic effects of the proposed components, a comprehensive ablation study was conducted. We systematically evaluated all valid combinations of the three key modules—C3k2_DRB, C2PSA_EPGO, and DySample—integrated into the baseline YOLOv11n model. The models were quantitatively evaluated using Precision (P) and mean Average Precision at IoU 0.5 (mAP@0.5) as the primary metrics. The detailed results of all 7 configurations are summarized in Table 4.

**Table 4:** Comprehensive ablation study on the effectiveness of the proposed modules.

Number	Baseline	C3k2_DRB	C2PSA_EPGO	DySample	Precision	mAP@0.5
1	✓				95.2	98.1
2	✓	✓			91.4	98.7
3	✓		✓		95.4	99.2
4	✓			✓	94.3	98.4
5	✓	✓	✓		96.9	99.1
6	✓	✓		✓	94.6	98.9
7	✓	✓	✓	✓	97.6	99.2

#### Analysis of results

Single-module configurations (Exps. 2–4). Introducing C3k2_DRB alone (Exp. 2) increases mAP@0.5 from 98.1 to 98.7% but causes a sharp drop in Precision from 95.2 to 91.4%. This is because the expanded receptive field from dilated reparameterization, while beneficial for multi-scale tumor recall, also increases false positives on non-lesion structures (e.g., vessels, airways) in the absence of a dedicated gating mechanism. In contrast, C2PSA_EPGO alone (Exp. 3) slightly improves Precision to 95.4% and substantially raises mAP@0.5 to 99.2%, demonstrating its effectiveness in suppressing background noise. DySample alone (Exp. 4) yields modest improvements in mAP@0.5 (98.4%) but reduces Precision (94.3%), suggesting its primary role is in boundary refinement rather than false positive suppression.

Pairwise configurations (Exps. 5–6). Critically, the combination of C3k2_DRB and C2PSA_EPGO (Exp. 5) restores Precision to 96.9% while maintaining high mAP@0.5 (99.1%), confirming the synergistic effect: C3k2_DRB provides broad multi-scale coverage, and C2PSA_EPGO prunes spurious background activations. The combination of C3k2_DRB and DySample (Exp. 6) recovers Precision only to 94.6%, indicating that DySample alone cannot compensate for the false positives introduced by the dilated reparameterization without the attention mechanism.

Full configuration (Exp. 7). Integrating all three modules achieves the best overall performance (Precision: 97.6%, mAP@0.5: 99.2%), validating that each module contributes complementary benefits: C3k2_DRB for multi-scale receptive fields, C2PSA_EPGO for discriminative noise suppression, and DySample for precise boundary alignment. The final model significantly outperforms the baseline and all partial combinations, demonstrating the necessity and synergy of the proposed architectural enhancements.

## Conclusion

To address challenges in lung cancer diagnosis, such as drastic tumor scale variations and complex backgrounds, this paper proposes an end-to-end fine-grained detection algorithm based on an improved YOLO architecture. Overcoming the limits of binary classification, our model achieves a complex 7-class detection task encompassing normal tissue, non-small cell lung cancer (NSCLC) subtypes, and specific TNM clinical stages. Specifically, we design a C3k2_DRB module to enlarge the receptive field via multi-scale dilated reparameterization. We also introduce the C2PSA_EPGO attention mechanism to suppress background noise and focus on discriminative lesion textures. Furthermore, a DySample strategy is employed for content-aware spatial alignment, preserving irregular morphological boundaries. Experimental results on 1886 images demonstrate that our model achieves 97.6% Precision and 99.2% mAP@0.5 with highly efficient computational costs (2.46 M parameters, 6.3 GFLOPs). Ultimately, this study serves as a methodological proof-of-concept, highlighting how targeted architectural enhancements can handle fine-grained and multi-scale challenges in medical image analysis. While the proposed architectural improvements demonstrate promising performance, we acknowledge several limitations in this study. First, the dataset utilized originates from an open-source platform and lacks rigorous clinical validation. The exceptionally high predictive performance (> 95% accuracy) reported in this study is likely heavily influenced by the specific characteristics, annotation biases, and homogeneous device artifacts inherent to this dataset. Furthermore, as a single 2D CT slice cannot definitively ascertain complete TNM staging or precise histological subtyping in real-world clinical practice, this framework must be viewed strictly as a computational proof-of-concept for handling complex, multi-class visual tasks. Readers should avoid conflating the algorithmic benchmark performance achieved here with true clinical diagnostic accuracy. Future work must involve validating this architectural framework on large-scale, multi-center cohorts with 3D volumetric data and board-certified clinical annotations to fully assess its true generalization capability and real-world deployability.

## Data Availability

The datasets used and/or analyzed during the current study are available from the corresponding author on reasonable request.
